# Role of Nutrition Education in Pharmacy Curriculum—Students’ Perspectives and Attitudes

**DOI:** 10.3390/pharmacy9010026

**Published:** 2021-01-23

**Authors:** Majid Mufaqam Syed-Abdul, Syed Sadath Kabir, Dhwani Satishkumar Soni, Tony J. Faber, Jeremy T. Barnes, Maureen T. Timlin

**Affiliations:** 1 Department of Child and Family Studies, Southeast Missouri State University, Cape Girardeau, MO 63701, USA; afaber@semo.edu; 2 Department of Kinesiology, Nutrition, and Recreation, Southeast Missouri State University, Parker Hall, MS7650, Cape Girardeau, MO 63701, USA; dhwani_soni4890@yahoo.in (D.S.S.); jbarnes@semo.edu (J.T.B.); 3 Department of Chemistry, Northeastern Illinois University, Chicago, IL 60625, USA; syedsadathkabir@gmail.com; 4 Department of Family Consumer Science, Minnesota State University, Mankato, MN 56001, USA; molly.timlin@mnsu.edu

**Keywords:** pharmacy, nutrition, education, attitude, course

## Abstract

Many pharmacists report they lack nutritional knowledge and believe the best time to educate pharmacists about nutrition is during pharmacy school. Purpose: This study was conducted to determine if today’s pharmacy students receive education in nutrition and if they realize the importance of a nutrition course. Methods: Ninety-five pharmacy students attending pharmacy school were surveyed in two pharmacy schools in the United States. Results: The survey showed only 13.7% received nutrition education and 82.9% of students believed nutrition education should be incorporated into the pharmacy degree curriculum. When the pharmacy-related experience was taken into account, 73.3% of students believed that a nutrition course should be incorporated into the curriculum. Conclusion: This study suggests that pharmacy students from two major universities in Alabama and Illinois realize the importance of nutrition education and believe a nutrition course should be incorporated into the pharmacy degree curriculum.

## 1. Specific Contribution to Special Edition

Concerns regarding the lack of nutrition knowledge among pharmacists have been raised previously. However, no studies have been conducted to see how many students take a nutrition course. Results from the current study report that only 1/10th of the pharmacy students receive nutrition education, and regardless of previous nutrition education or pharmacy-related experience, pharmacy students believed that pharmacy schools should incorporate nutrition education. Although data from only two schools are reported here, this protocol sets the stage for future studies to conduct an analysis of nutrition education among pharmacy students from other schools and their perspective towards adding a nutrition course in the pharmacy degree curriculum.

## 2. Introduction

Pharmacists have been traditionally regarded as (and regard themselves as) experts in medications, not in food and nutrition [1]. However, in recent years pharmacists have realized their importance not only in controlling adverse food–drug interactions, but also in in-patient counseling [2,3], and health education [4]. Additionally, pharmacists have taken on the role of public healthcare providers/advisors for chronic conditions such as for overweight/obesity, diabetes [5], under-nutrition [6], and parenteral nutrition support [7]. Today, pharmacists are more accessible to the general public [1,5,8,9,10], in-part because many pharmacies are now also located in grocery stores [8]. This allows the general public to access pharmacists during their grocery shopping, saving an additional trip to the pharmacy. Because one-third of products sold in pharmacies are nutritional products or nutraceuticals [5], it has become necessary for pharmacists in today’s market to have knowledge of nutrition [9]. Additionally, due to the increase in the number of pharmacies and accessibility of pharmacists in the United States, pharmacists will likely encounter questions not only about prescription drugs but also about nutritional products. Furthermore, definite links between diets and diseases are becoming evident, which means it is extremely important for pharmacists to prepare themselves with the necessary knowledge in the field of nutrition [11]. Many pharmacists have come to realize they do not possess enough knowledge in the discipline of nutrition [12,13]. However, whether pharmacy graduates of today are well prepared to accept these new challenges and roles is still unknown. Therefore, the purpose of this study was to determine the number of pharmacy students who have taken a nutrition course and to recognize the importance of nutrition courses among pharmacy students. For the purpose of this study, a nutrition course was defied as the course that was formally included the word “nutrition” in the title of the course. The second purpose was to investigate how factors such as past nutrition education and pharmacy-related experience influence their opinion on the need for a basic nutrition course in a pharmacy degree core curriculum.

## 3. Methods

*Study population*: The study was approved by the College of Health and Human Services Committee for Research on Human Subjects at Southeast Missouri State University.

The schools were selected randomly, and prior approval for distribution of the questionnaire was received by contacting each university dean through email. Upon approval by each dean, an email containing a link to the questionnaire was forwarded by the researcher to the deans. The total duration of PharmD is four years after pre-pharmacy or a baccalaureate degree. Therefore, to select senior students, the deans then forwarded survey email to the pharmacy students enrolled in the third and fourth year of their respective PharmD program. A total of 95 students completed the survey. Pharmacy schools that were surveyed included Midwestern University, Chicago College of Pharmacy (MWUCCP), IL, and Harrison School of Pharmacy, Auburn University (HSPAU), AL. Fourteen other pharmacy schools were contacted but were not approved to conduct a survey or did not respond to the initial email.

*Instrumentation:* The survey was developed by the researchers from the Departments of Kinesiology, Nutrition, and Recreation, and Child and Family Studies, at Southeast Missouri State University. Three demographic questions and six questions specific to the study were included in the questionnaire. The following research questions were answered in this study:

1. What percentage of current pharmacy students take a course in nutrition?
2. What percentage of pharmacy students stated the need or importance of a nutrition course in their curriculum?
3. What percentage of pharmacy students stated their pharmacy schools required a nutrition course?
4. Does past education in nutrition affect the attitudes of pharmacy students towards a nutrition course?
5. Does past pharmacy-related experience affect the attitudes of pharmacy students towards a nutrition course?

*Data Collection:* Data was collected for five months by the use of SurveyMonkey.com. Informed consent was displayed as the first part of the survey followed by the questionnaire.

*Statistical* Procedures: Statistical Package for the Social Science v.21 (SPSS, IBM Corp., Armonk, NY, USA) was used to analyze the data. The research questions were answered using the data analysis methods of descriptive statistics and cross-tab analysis.

*Treatment of Missing* Data: The survey was set up in a way that students would not be able to submit their responses until they completed all the required questions. As a result of this, there were no missing data for the required questions. Participants’ responses were included even if they missed demographic related questions (Q 7–9).

## 4. Results

Demographics: As shown in Table 1, subjects were approximately 24% male (*n* = 23) and 76% female (*n* = 72). The majority of the subjects reported themselves as white or caucasian (76%) and were between the ages of 18–24 (54%, *n* = 51) and 25–34 years (41%, *n* = 39).

Education Status: Research Question 1—What percentage of current pharmacy students take a course in nutrition?

**Figure 1:**
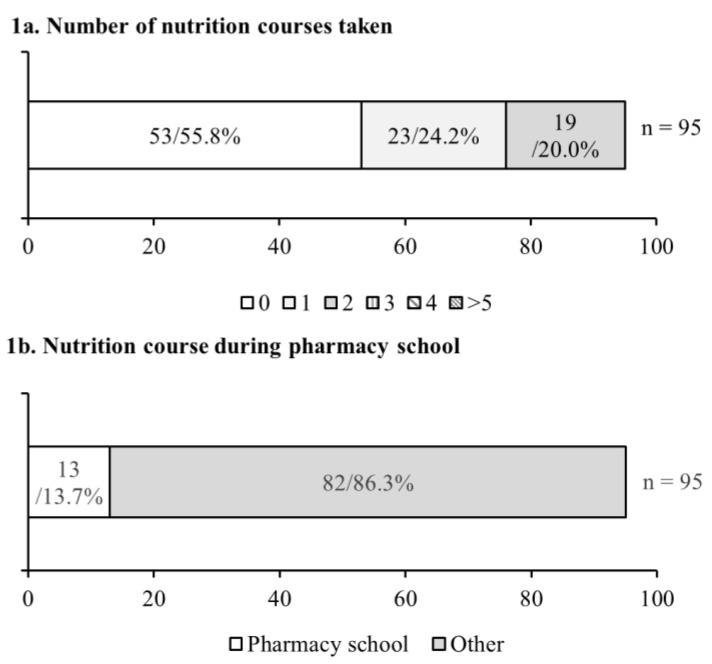
A total number of nutrition courses taken during pharmacy education. (**a**) Nutrition courses taken while in pharmacy school. (**b**) The combined number of nutrition courses taken before and during pharmacy school. The first number in each bar represents the number of students (*n*), the second represents the percent of the total. The *X*-axis represents n.

To determine how well the pharmacy students were educated in the discipline of nutrition, students were asked how many basic nutrition courses they had taken and when the course was taken. For this study, students who took two or more basic nutrition courses were considered well educated. As shown in Figure 1a, 20% (*n* = 19) of the students stated they had taken two or more nutrition courses. Only 13.7% (*n* = 13) of the students stated they had taken it during pharmacy school (Figure 1b). It was also found that 55.79% (*n* = 53) of students did not have any nutrition education (Figure 1a).

Importance of Nutrition Course: Research Question 2—What percentage of pharmacy students stated the need or importance of a nutrition course in their curriculum?

**Figure 2:**
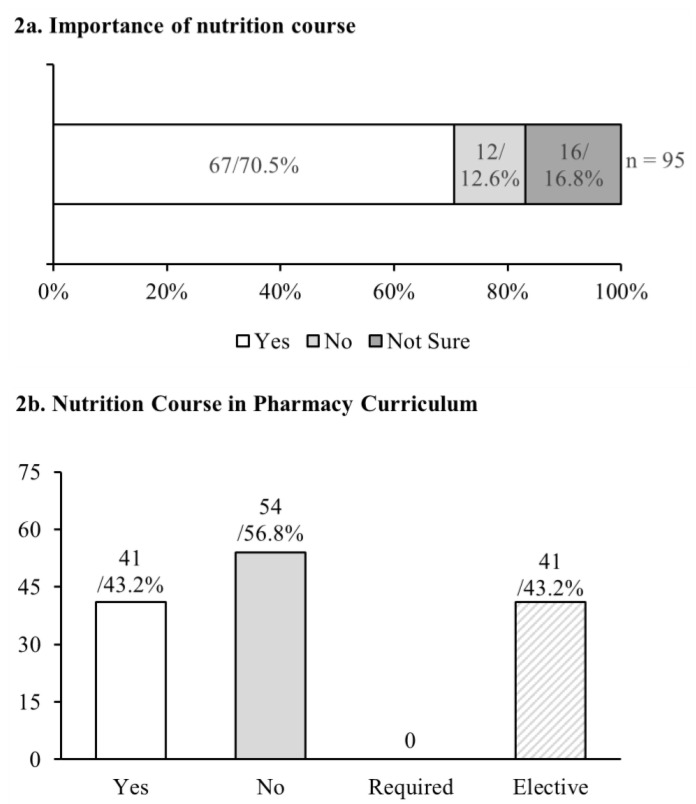
Students’ perspectives on the importance of a nutrition course and if the course was offered in their pharmacy school. (**a**) Didf students feel the nutrition course was important during their pharmacy education? (**b**) Did students feel the nutrition course was offered in their pharmacy curriculum? If yes, whether it was required or elective. The first number for each bar represents the number of students (*n*), and the second represents the percent of the total.

A total of 70.5% (*n* = 67) of the students felt that a course in nutrition was an important component of education for the pharmacist (Figure 2a). Twelve (12.6%) students reported it is not an important component of the pharmacy curriculum, and sixteen 16 (16.8%) were unsure.

Nutrition Course in Pharmacy Curriculum: Research Question 3—What percentage of pharmacy students stated their pharmacy schools required a nutrition course?

As shown in Figure 2b, a total of 43.1% (*n* = 41) of students stated that their pharmacy schools offered a nutrition course. However, none of the students who stated that their pharmacy school offered a nutrition course stated it was a required course, i.e., it was offered as an elective course. Further, 56.8% (*n* = 54) of students reported that their schools did not offer any nutrition course.

The Attitude of Pharmacy Students: Research Question 4—Does past education in nutrition affect the attitudes of pharmacy students towards a nutrition course? 

**Figure 3:**
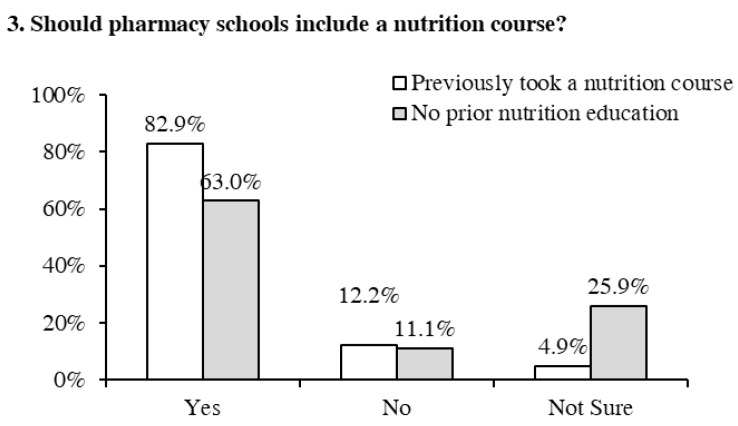
Previous nutrition education and students’ perspectives on adding a nutrition course in the pharmacy degree curriculum.

As shown in Figure 3, among the students who took a nutrition course, 82.9% (*n* = 34) of them stated that pharmacy schools should include a nutrition course in the pharmacy curriculum. Interestingly, 63% (*n* = 34) of the students who had not taken a nutrition course in their pharmacy curriculum also had a positive attitude towards the need for this course. Approximately 12% of students (regardless of prior nutrition education) reported that pharmacy schools should not include a nutrition course in the curriculum. Lastly, around 5% of students who had taken nutrition courses in the past and 26% of the students who did not have any prior nutrition education reported that they were unsure.

Research Question 5—Does past pharmacy-related experience affect the attitudes of pharmacy students towards a nutrition course?

**Figure 4:**
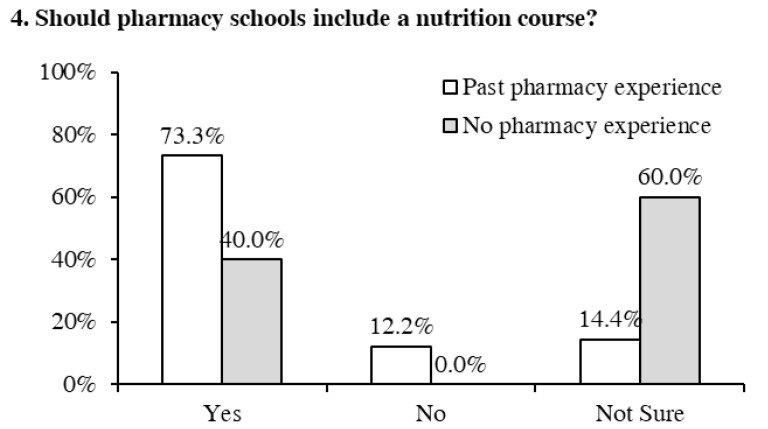
Previous pharmacy-related experience and students’ perspectives on adding a nutrition course in the pharmacy degree curriculum.

In addition to past nutrition education, we also investigated how past pharmacy-related experience influenced the attitudes of the pharmacy students towards the need for a course in nutrition. A total of 93.7% (*n* = 89) of the students stated they had the pharmacy-related experience. Among these students, as shown in Figure 4, 73.3% (*n* = 66) of the students thought that pharmacy schools should include a nutrition course in the pharmacy curriculum. Moreover, 40% (*n* = 2) of the students who did not have any experience also showed a positive attitude towards the need for a nutrition course. Twelve percent of students with pharmacy experience reported they felt a nutrition course should not be included. Approximately 14% of students with pharmacy experience and 60% (*n* = 3) of students without prior pharmacy experience reported that they were unsure if the nutrition course should be incorporated in the pharmacy degree curriculum.

## 5. Discussion

The main purpose of this study was to investigate the perceptions of pharmacy students towards incorporating a nutrition course in the pharmacy degree curriculum. Results of this study document that the majority of pharmacy students felt a course in nutrition is important and believe it should be incorporated in the pharmacy degree curriculum. These results are consistent with a recent study conducted on community pharmacists [12]. In the current study, 43.2% of pharmacy students stated that they had taken a nutrition course but it was taken as an elective. Neither of the pharmacy schools surveyed requires completion of a nutrition course as part of the pharmacy curriculum. These outcomes are consistent with the results from a previous study where it was reported that few pharmacy schools incorporate a nutrition course as an elective [11]. The current study documented that both of the pharmacy schools in the AL and IL did not offer nutrition as a core course in the curriculum.

Current Education Status: Past literature suggests that pharmacists’ have been lacking nutrition education. This issue was identified more than two decades ago [13]. It is surprising that this issue still exists and in fact that nutrition education is getting worse among pharmacists and other healthcare professionals [14]. Findings from our study are consistent with previous studies. The vast majority of pharmacy students do not get enough nutrition education and are not well educated within the area of nutrition [11]. In the current study, 80% reported they were not well educated in the field of nutrition (i.e., they had not taken two or more nutrition courses) and only 14.7% of the students had taken two nutrition courses during pharmacy school. Additionally, only 24.2% of students stated they had taken only one nutrition course. Whether this course prepared them well to be able to answer a nutrition-related question is still unknown. As indicated by Boggs et al. [13], more than two decades ago, pharmacists were dealing with the same issues in the past which needs an attention.

Nutrition Course in Pharmacy Curriculum: Currently, there are no core nutrition courses offered in the pharmacy schools that were surveyed in this study. These results are consistent with Miller’s study [11], who documented that the majority of pharmacy schools do not require a nutrition course in the pharmacy degree curriculum. Given that the study conducted by Miller et al. is not current, a new investigation of pharmacy schools is needed to better understand the current status of nutrition education in the pharmacy curriculum. However, given the responses obtained from the two pharmacy schools, the situation appears to be the same even today. It is a crucial time point for pharmacy schools to consider incorporating a core nutrition course in their curriculum to prepare future pharmacists with all the future challenges.

The Attitude of Pharmacy Students towards Incorporating a Nutrition Course: The majority of the students whose pharmacy school did not offer a nutrition course as well as those students whose pharmacy school did offer a nutrition course in their curriculum believed a nutrition course should be incorporated into the pharmacy degree curriculum. Interestingly, these responses appeared to be influenced by students’ past pharmacy-related experience. Of students who had pharmacy-related experience, 73.3% stated that a nutrition course should be included in the pharmacy degree curriculum. A possible explanation for these findings may be that these individuals have been asked about nutrition-related questions through their work in a pharmacy setting [15]. Similar attitudes were observed among the pharmacists as well who felt that nutrition education is crucial for their practice in community pharmacies [16]. This is perhaps because of pharmacists’ accessibility, nutritional products sold in pharmacies, and more importantly the increasing evidence of definite links between diets and diseases [1,5,8,9,10,11]. These data suggest that it is extremely important for pharmacy schools to prepare pharmacists with the necessary nutrition knowledge for upcoming challenges.

Limitations: In this study, a strong effort was taken to control as many variables as possible; however, there are a few limitations that need to be discussed. The study was conducted only in two pharmacy schools, and the sample size was small (*n* = 95). Therefore, results from this study may be limited to the study population as results may not be representative of all pharmacy schools in the United States. The other limitation of this study is that the information on the pharmacy curriculum was obtained from students rather than the school’s websites.

## 6. Conclusions

Consistent with previous literature, this study documented that today’s pharmacists lack nutrition education. Both experienced and inexperienced students realize the importance of nutrition education and have started to educate themselves by taking it as an elective course; additionally, most current pharmacists believe the best time for a course in nutrition is during pharmacy school. Some schools have begun to incorporate nutrition content into current courses but the results from this study document that the pharmacy schools offer a core (i.e., required) nutrition course.

*Future Studies and Recommendations*: More pharmacy students from the United States and other countries should be surveyed to gain a better understanding of the attitude of pharmacy students towards a nutrition course.

## Figures and Tables

**Table 1:** Demographics.

	United States (*n* = 95)
	*n*/%
**Gender**	
Male	23/24.2
Female	72/75.8
**Age**	
18–24 years	51/53.7
25–34 years	39/41.1
35–44 years	3/3.2
> 45 years	2/2.1
**Ethnicity**	
African American	1/1.1
Hispanic/Latino	0/0
Mexican	1/1.1
Indian	6/6.3
Caucasian/white	72/75.8
Multiracial	5/5.3
Other	10 */10.5

* 6 Asians, 2 Indians born in the US, 1 Arab (Middle Eastern), and 1 Pakistani born in the US.

## Data Availability

The data presented in this study are available on request from the corresponding author.
